# Acute Carpal Tunnel Syndrome Secondary to Handcuffs Necessitating Emergency Orthopedic Consultation and Operative Intervention

**DOI:** 10.1016/j.acepjo.2024.100013

**Published:** 2025-01-10

**Authors:** Fabian Jano, Kelly MacKenzie, Vivek K. Bilolikar, David Goldberger, Andrei Tuluca

**Affiliations:** 1 Department of Emergency Medicine, Emergency Medicine Residency Program, Jefferson Einstein Philadelphia Medical Center, Philadelphia, Pennsylvania, USA; 2 Sidney Kimmel Medical College, Thomas Jefferson University, Philadelphia, Pennsylvania, USA; 3 Department of Orthopedic Surgery, Orthopedic Surgery Residency Program, Jefferson Einstein Philadelphia Medical Center, Philadelphia, Pennsylvania, USA

**Keywords:** acute carpal tunnel syndrome, carpal tunnel syndrome, handcuff injury, handcuff neuropathy, median nerve injury, radial nerve injury

## Abstract

We present a case of acute carpal tunnel syndrome secondary to tight handcuffs in a detained patient. The severity of the motor and sensory deficits prompted consultation with orthopedic specialists and admission for an observation period with ultimate emergency operative intervention. Handcuff neuropathies are well documented in the literature, mainly involving the superficial branch of the radial nerve, which has strict sensory input into the dorsum of the hand. Less common median nerve neuropathies have also been documented following handcuff injury; however, none have required emergency intervention. We aim to highlight this unusual presentation and the emergency nature of the condition, as well as advocate for increased caution when caring for detained patients in the emergency department.

## 1 Introduction

Carpal tunnel syndrome (CTS) is the most common entrapment neuropathy. The median nerve is contained within the carpal tunnel; increased compartmental pressures result in nerve dysfunction and the development of CTS. Long-term increased pressures lead to local nerve ischemia, releasing inflammatory factors that cause edema and further increased pressures within the carpal tunnel. Chronic CTS is generally an idiopathic condition but is thought to be related to occupational exposure involving hand gripping, repetitive flexion and extension of the wrist, and vibration. Initially, the sensory fibers of the median nerve are affected, with later-stage involvement of motor fibers. The condition can also be secondary to a myriad of primary processes, such as pregnancy, obesity, hypothyroidism, diabetes, congestive heart failure, and genetic predisposition. Symptomatology includes initial nocturnal paresthesia involving the thumb, index, middle, and radial half of the ring finger with sparing of the thenar eminence, progressing to daytime symptoms and eventual atrophy of the thenar eminence.^1^

Acute CTS (ACTS) is caused by a sudden increase in carpal tunnel pressure, most commonly secondary to wrist trauma. Increased carpal tunnel pressure in these cases is usually due to hemorrhage, edema, or direct trauma from bone fragments. Distal radial fractures are the most common traumatic mechanism causing ACTS. The incidence of ACTS following distal radius fractures is 5.4% to 8.6%. ACTS caused by distal radius fractures is progressive and does not improve without operative intervention. In contrast, nerve neuropraxia, which is caused by nerve contusion and stretch injury from distal radius fractures, resolves with rest and time. ACTS necessitates immediate intervention to reduce pressure on the median nerve and the potential need for carpal tunnel release.^2^^,^^3^

ACTS is not well reported in the literature following handcuff trauma. A systematic review by Khan et al^4^ of handcuff injuries revealed more superficial neuropathies, specifically superficial branches of the radial nerve, as a common complication of handcuffs. Median nerve neuropathy was also noted, with only 1 case documenting mild ACTS as the inciting mechanism of the neuropathy.^4^^,^^5^ We present a case of ACTS caused by tight handcuffs, necessitating emergency orthopedic consultation for carpal tunnel release. To our knowledge, this is the first documented case of severe median nerve injury from handcuffs.

## 2 Case Presentation

The patient is a 24-year-old right-hand dominant male with no significant past medical or surgical history who presented to the emergency department with a chief complaint of right wrist and hand pain. The patient was in the custody of police with handcuffs in place, with additional complaints of handcuffs being very tight. Symptoms began shortly after handcuff placement, with associated paresthesia of the entire dorsum of the hand and inability to move the thumb and index fingers. There was significant struggle and resistance reported during the arrest process. There was no improvement in his symptoms following the loosening of his handcuffs within 30 minutes of placement. He otherwise denied any antecedent symptoms or any history of nerve injuries. There was no other trauma reported prior to the arrest.

On examination, the patient had unremarkable vital signs and appeared to be in pain. There was notable skin indentation with erythema from handcuffs at the right wrist. He had tenderness to palpation of the right proximal palm and thenar eminence with decreased pinprick and 2-point discrimination in the median and radial nerve distribution. The patient was unable to flex the thumb at the metacarpophalangeal and interphalangeal joints, adduct, and oppose the thumb against any of the other digits. Additionally, the patient was unable to flex the index finger at the metacarpophalangeal, proximal interphalangeal, and distal interphalangeal joints. There was weak finger abduction globally and a positive Tinel sign. Pulses were intact, and there was no forearm or hand compartment rigidity. Wrist strength and the rest of ipsilateral upper-extremity strength testing were normal. X-ray imaging of the hand and wrist did not reveal fractures, signs of ligamentous disruption, or dislocations.

The differential diagnosis included ACTS, superficial radial nerve neuropathy, median and superficial radial nerve neurapraxia, occult forearm and wrist fracture or dislocation, compartment syndrome, and unlikely brachial plexus injury. Due to persistent neurologic deficits in the emergency department that did not improve with the removal of handcuffs or pain control, orthopedic surgery was consulted. The orthopedic team recommended placing the right hand in a volar slab splint and admitting him to the observation unit for frequent neurologic checks. Given no improvement in his deficits, he was taken for emergency operative median nerve exploration and carpal tunnel release within 4 hours of admission, 8 hours since the initial presentation to the emergency department. The operative report noted significant neuronal edema of the median nerve extending proximally from the carpal tunnel.

The patient tolerated the procedure well, and strength and sensation in his thumb and index finger gradually returned postoperatively. He was discharged on postoperative day zero. At his 2-week postoperative appointment, the patient had near resolution of symptoms within the median nerve distribution, with residual mild sensory deficits in the radial nerve distribution.

## 3 Discussion

Handcuffs work by deploying a ratchet mechanism, allowing only further tightening until unlocked. This mechanism is vulnerable to inadvertent overtightening in scenarios where there is significant struggle and resistance by the subject or abrupt closure by law enforcement officers.^6^ There was significant struggle reported during the arrest process in our case, followed by up to 30 minutes of sustained pressure/trauma caused by the handcuffs. We aim to highlight and raise awareness of this vulnerability in the mechanism that typical handcuffs deploy and how it can lead to significant morbidity.

Neuropathies caused by handcuffs are well documented in the literature.^4^ Namely, the superficial branch of the radial nerve is susceptible to compression injury due to its superficial course over the distal radius.^5^ Grant et al^6^ performed a prospective study analyzing wrist injuries from handcuffs and found that radial nerve injury was present in 81% of handcuff neuropathies; injuries varied from complete loss of nerve function to persistent paresthesias. Our patient did have sensory deficits and paresthesia in the superficial radial nerve distribution, which is consistent with compression injury. Notably, external signs of compression, such as hand edema, ecchymosis, abrasions, and lacerations, were present in 56% of the subjects in the study, further indicating the necessity of a comprehensive neurologic examination to identify any deficits in the superficial radial nerve distribution as an indicator of compression injury.^6^

Median nerve neuropathies from handcuffs are also noted in the literature.^4^, ^5^, ^6^, ^7^, ^8^ As opposed to the purely sensory function of the superficial radial nerve, median nerve injury imposes significant disability given its motor function.^7^^,^^9^ The systematic review of handcuff injuries by Khan et al^4^ revealed that 39% (19/49) had involvement of the median nerve in their population of handcuff neuropathies. Only 1 study by Dorfman et al^5^ attributed the cause to ACTS, although it was mild without any motor deficit and was treated with a splint. Only 1 case by Haddad et al^10^ was followed by operative exploration for median nerve injury without any elaboration on the specific procedure performed, findings, and outcomes. Therefore, our case is unique in that it is the first documented case of severe injury to the median nerve from handcuffs, without any improvement during a brief observation period, followed by rapid resolution of deficits status post carpal tunnel release and neuroplasty.

Incarcerated patients are a particularly vulnerable population who require specific attention from the clinician. Notably, they are more likely to experience dismissal of health complaints, delayed hospital presentation, and lack of access to treatment.^11^ It is essential to note the detrimental effect of clinician bias on clinical outcomes in this patient population, as it often leads to incomplete workups and therapeutic plans.^12^ This is especially pertinent to the emergency medicine physician as much of the health care received by patients in the custody of police begins in the emergency department. Ultimately, it would be prudent to consider the health care disparities incarcerated patients experience and to thoroughly investigate all their medical complaints to ensure they receive appropriate evaluation and treatment, as demonstrated in our case.

## Funding and Support

By *JACEP* *Open* policy, all authors are required to disclose any and all commercial, financial, and other relationships in any way related to the subject of this article as per ICMJE conflict of interest guidelines (see www.icmje.org). The authors have stated that no such relationships exist.
